# The Normal Range of Baseline Tryptase Should Be 1 to 15 ng/mL and Covers Healthy Individuals With HαT

**DOI:** 10.1016/j.jaip.2023.08.008

**Published:** 2023-08-10

**Authors:** Peter Valent, Gregor Hoermann, Patrizia Bonadonna, Karin Hartmann, Wolfgang R. Sperr, Sigurd Broesby-Olsen, Knut Brockow, Marek Niedoszytko, Olivier Hermine, Yannick Chantran, Joseph H. Butterfield, Georg Greiner, Melody C. Carter, Vito Sabato, Deepti H. Radia, Frank Siebenhaar, Massimo Triggiani, Theo Gülen, Ivan Alvarez-Twose, Thomas Staudinger, Ludwig Traby, Karl Sotlar, Andreas Reiter, Hans-Peter Horny, Alberto Orfao, Stephen J. Galli, Lawrence B. Schwartz, Jonathan J. Lyons, Jason Gotlib, Dean D. Metcalfe, Michel Arock, Cem Akin

**Affiliations:** a Division of Haematology, Department of Internal Medicine I, Medical University of Vienna, Vienna, Austria; b Ludwig Boltzmann Institute for Hematology and Oncology, Medical University of Vienna, Vienna, Austria; c MLL Munich Leukemia Laboratory, Munich, Germany; d Allergy Unit, Verona University Hospital, Verona, Italy; e Division of Allergy, Department of Dermatology, University Hospital Basel and University of Basel, Basel, Switzerland; f Department of Biomedicine, University Hospital Basel and University of Basel, Basel, Switzerland; g Department of Dermatology and Allergy Centre, Odense University Hospital, Odense, Denmark; h Department of Dermatology and Allergy Biederstein, Technical University of Munich, Munich, Germany; i Department of Allergology, Medical University of Gdansk, Gdansk, Poland; j Service d’hématologie, Imagine Institute Université de Paris, Centre national de référence des mastocytoses, Hôpital Necker, Assistance publique hôpitaux de Paris, Paris, France; k Department of Biological Immunology, Saint-Antoine Hospital, Paris Sorbonne University, Paris, France; l Division of Allergic Diseases, Mayo Clinic, Rochester, Minn; m Ihr Labor, Medical Diagnostic Laboratories, Vienna, Austria; n Laboratory of Allergic Diseases, National Institute of Allergy and Infectious Diseases (NIAID), National Institutes of Health (NIH), Bethesda, Md; o Faculty of Medicine and Health Sciences, Department of Immunology-Allergology-Rheumatology, University of Antwerp and Antwerp University Hospital, Antwerp, Belgium; p Guy’s & St. Thomas’ National Health Service (NHS) Foundation Trust, Guy’s Hospital, London, UK; q Institute of Allergology, Charité-Universitätsmedizin Berlin, Corporate Member of Freie Universität Berlin, Humboldt-Universität zu Berlin, and Berlin Institute of Health, Berlin, Germany; r Fraunhofer Institute for Translational Medicine and Pharmacology (ITMP), Immunology and Allergology (IA), Berlin, Germany; s Division of Allergy and Clinical Immunology, University of Salerno, Salerno, Italy; t Department of Respiratory Medicine and Allergy, Karolinska University Hospital Huddinge, Stockholm, Sweden; u Department of Medicine Solna, Division of Immunology and Allergy, Karolinska Institutet, Stockholm, Sweden; v Instituto de Estudios de Mastocitosis de Castilla La Mancha (CLMast) and CIBERONC, Hospital Virgen del Valle, Toledo, Spain; w Department of Internal Medicine I, Intensive Care Unit, Medical University of Vienna, Vienna, Austria; x Department of Internal Medicine I, Division of Infectious Diseases and Tropical Medicine, Medical University of Vienna, Vienna, Austria; y Institute of Pathology, University Hospital Salzburg, Paracelsus Medical University, Salzburg, Austria; z Department of Hematology and Oncology, University Hospital Mannheim, Mannheim, Germany; aa Institute of Pathology, Ludwig-Maximilians-University, Munich, Germany; bb Servicio Central de Citometria, Centro de Investigacion del Cancer (IBMCC CSIC/USAL) Instituto Biosanitario de Salamanca (IBSAL), CIBERONC and Department of Medicine, University of Salamanca, Salamanca, Spain; cc Department of Pathology, Department of Microbiology and Immunology, Sean N. Parker Center for Allergy and Asthma Research, Stanford University School of Medicine, Stanford, Calif; dd Department of Internal Medicine, Division of Rheumatology, Allergy, and Immunology, Virginia Commonwealth University, Richmond, Va; ee Translational Allergic Immunopathology Unit, Laboratory of Allergic Diseases, NIAID, NIH, Bethesda, Md; ff Stanford University School of Medicine/Stanford Cancer Institute, Stanford, Calif; gg Department of Hematological Biology, Pitié-Salpêtrière Hospital, Paris Sorbonne University, Paris, France; hh Division of Allergy and Clinical Immunology, University of Michigan, Ann Arbor, Mich

**Keywords:** Tryptase, Mastocytosis, Hereditary alpha tryptasemia, Mast cell activation syndromes

## Abstract

Physiological levels of basal serum tryptase vary among healthy individuals, depending on the numbers of mast cells, basal secretion rate, copy numbers of the *TPSAB1* gene encoding alpha tryptase, and renal function. Recently, there has been a growing debate about the normal range of tryptase because individuals with the hereditary alpha tryptasemia (HαT) trait may or may not be symptomatic, and if symptomatic, uncertainty exists as to whether this trait directly causes clinical phenotypes or aggravates certain conditions. In fact, most HαT-positive cases are regarded as asymptomatic concerning mast cell activation. To address this point, experts of the European Competence Network on Mastocytosis (ECNM) and the American Initiative in Mast Cell Diseases met at the 2022 Annual ECNM meeting and discussed the physiological tryptase range. Based on this discussion, our faculty concluded that the normal serum tryptase range should be defined in asymptomatic controls, inclusive of individuals with HαT, and based on 2 SDs covering the 95% confidence interval. By applying this definition in a literature screen, the normal basal tryptase in asymptomatic controls (HαT-positive persons included) ranges between 1 and 15 ng/mL. This definition should avoid overinterpretation, unnecessary referrals, and unnecessary anxiety or anticipatory fear of illness in healthy individuals.

## INTRODUCTION

Mast cells (MCs) are tissue-resident, immune-regulatory effector cells involved in the initiation and perpetuation of allergic inflammation as well as in repair after tissue injury and in several other physiological processes.^1–6^ In common with blood basophils, MCs express high-affinity immunoglobulin E (IgE)–binding sites (FcεRI) and store pro-inflammatory, vasoactive, and repair-supporting mediator substances in their meta-chromatic granules.^1–6^ During a severe anaphylactic reaction, allergen-induced cross-linking of FcεRI on MCs induces an explosive release of granule-associated mediators. In addition, activated MCs release newly formed, cell membrane–derived (lipid-type) mediators of hypersensitivity reactions as well as newly synthesized cytokines into the extracellular space.^1–6^ Blood basophils may also participate in allergies through FcεRI-dependent reactions by releasing a similar profile of mediators.^1,7,8^ However, not all hypersensitivity reactions may involve basophils, even if the reaction is systemic. Moreover, some of the relevant mediators and repair molecules are produced and released primarily by MCs but not by basophils.

The ability of MCs and basophils to liberate mediators of anaphylaxis in the context of cell activation, also known as releasability, depends on several factors, including the underlying (primary) disease/pathology, the numbers and type of activated surface receptors, the signaling pathways involved, and the genetic background.^3–15^ The clinical picture and severity of an anaphylactic reaction depends on additional variables, such as the number and anatomical location of MCs involved in the reaction, amount of mediators secreted, presence and type of allergens, and triggering cofactors such as temperature, exercise, alcohol, medicines, and/or comorbidities.^4–16^

Mast cell activation may occur in a number of pathological reactions. Acute MC activation is often seen in allergic (hypersensitivity) reactions and may be associated with symptoms and signs of anaphylaxis.^4–6,13–20^ Severe or even life-threatening events may occur when MCs are in a hyper-reactive state and/or the burden of MCs is high.^13–20^ In such patients, a mast cell activation syndrome (MCAS) may be diagnosed that is confirmed by documenting the presence of MCAS criteria.^21–24^ Historically, clinical symptoms arising from MC activation have primarily been studied in the context of allergic/atopic disorders.^3–6,11–15^ More recently, MC activation has also been analyzed in the context of other etiologies, including mastocytosis.^2,4,5,16–24^

**FIGURE 1.**
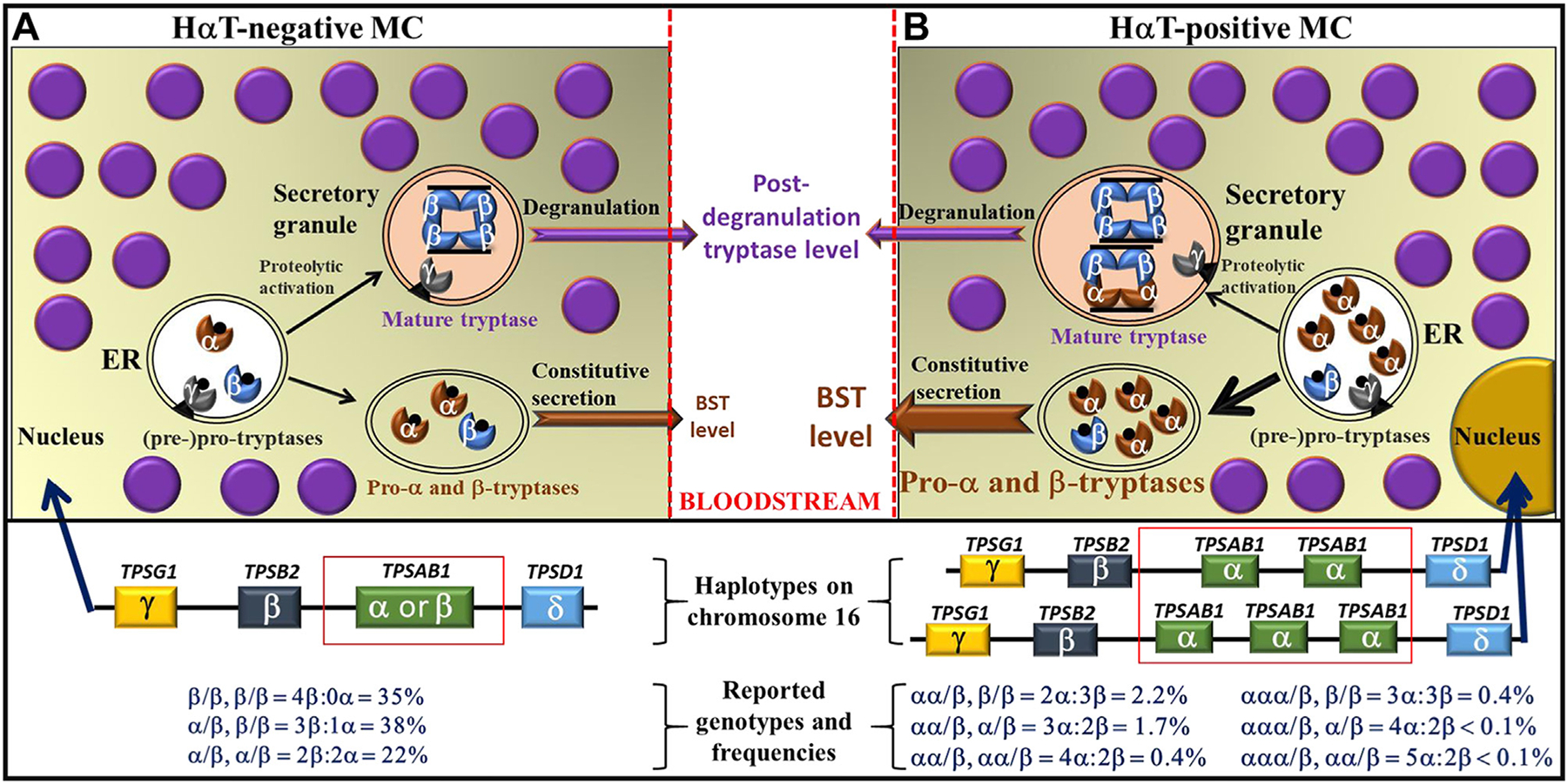
Synthesis of tryptases by MCs in (**A**) HαT-negative and (**B**) HαT-positive individuals. (**A**) In HαT-negative human MCs (~95% of the population), the tryptase locus (bottom) on each haplotype contains 4 genes encoding tryptase (*TPSG1*, *TPSB2*, *TPSAB1*, and *TPSD1*) on the short arm of chromosome 16 at p13.3. It is worth noting that, among all these genes, only *TPSB2* and *TPSAB1* encode for secreted (serum) tryptase isoforms. Whereas *TPSB2* encodes only beta-tryptase, *TPSAB1* encodes either alpha or beta isoforms.^29^ The resulting genotypes and their frequencies are presented in **A**. In MCs (**A**, top), tryptase is produced in alpha, beta, gamma, and delta subunits in the endoplasmic reticulum (ER).^29^ Although the gamma subunit is bound to granule membranes, alpha and beta monomers are continuously released as inactive pro-peptides from resting mast cells (without activation). Furthermore, beta subunits undergo sequential proteolytic cleavage (activation) to become a mature tetrameric tryptase that is stored in secretory granules of MCs (top) until degranulation occurs. (**B**) In HαT-positive MCs, the tryptase locus (bottom) on each haplotype still contains 1 copy of each *TPSG1*, *TPSB2*, and *TPSD1*, while the *TPSAB1* gene is duplicated, triplicated, or even more replicated, leading to different genotypes (bottom). This results in increased synthesis of pro-alpha tryptase (top) and an increased BST level compared with MCs in HαT-negative individuals. *BST*, basal serum tryptase; *HaT*, hereditary alpha tryptasemia; *MCs*, mast cells.

Tryptases are serine proteases that are primarily synthesized and stored in MCs, and less abundantly also in basophils.^25–28^ Several different forms of the enzyme have been described, including alpha and beta tryptases that contribute to measured levels in serum and other biological fluids.^25,26,28^ Whereas alpha and beta tryptase precursors are both stored and constantly released from resting MCs, mature tryptases are primarily stored in resting MCs and are only released during an anaphylactic event (Figure 1, A).^28,30–32^ Constitutive release of tryptases from MCs results in a basal serum tryptase (BST) level that is relatively stable in individual persons.^24,25,31^ However, the normal range of BST in the general population varies, depending on genetic features, comorbidities, and other factors.^31–37^ The most prevalent genetic condition that leads to an increase in the BST level is hereditary alpha-tryptasemia (HαT).^33–38^ Basal tryptase may also change during the life-time in individual patients, depending on renal function and acquired comorbidities.^33–37^

The normal range of BST has been under debate for many years based on the fact that the upper limit of normal varied (ranging from 8.2 ng/mL to 15 ng/mL) among the studies that were published in the past.^34–44^ So far, it remains unclear why different upper limits of normal values were obtained in healthy controls. One important aspect may be HαT, because this condition is often associated with an elevated tryptase level (Figure 1, B). In particular, the presence of HαT-positive individuals in a reference cohort leads to a bimodal distribution of serum tryptase levels, and the different portions of HαT-positive persons in these cohorts and differences in the applied statistical models may explain the observed variation in the upper limit of normal.

To discuss the normal range of BST in healthy individuals in light of recent developments in the field, including HαT, experts of the European Competence Network on Mastocytosis (ECNM) and the American Initiative in Mast Cell Diseases (AIM) met in Basel, Switzerland, in 2022. The resulting proposal is presented in the current manuscript.

This proposal includes a genetically determined normal upper tryptase limit of 8 ng/mL below which the likelihood of HαT is rather low. The clinically relevant cut-off for tryptase is higher (15 ng/mL) in order to include healthy individuals with HαT and all other controls, whereas in most patients with systemic mastocytosis (SM), tryptase levels are higher (>15 ng/mL).

### Causes of elevated levels of BST

**FIGURE 2.**
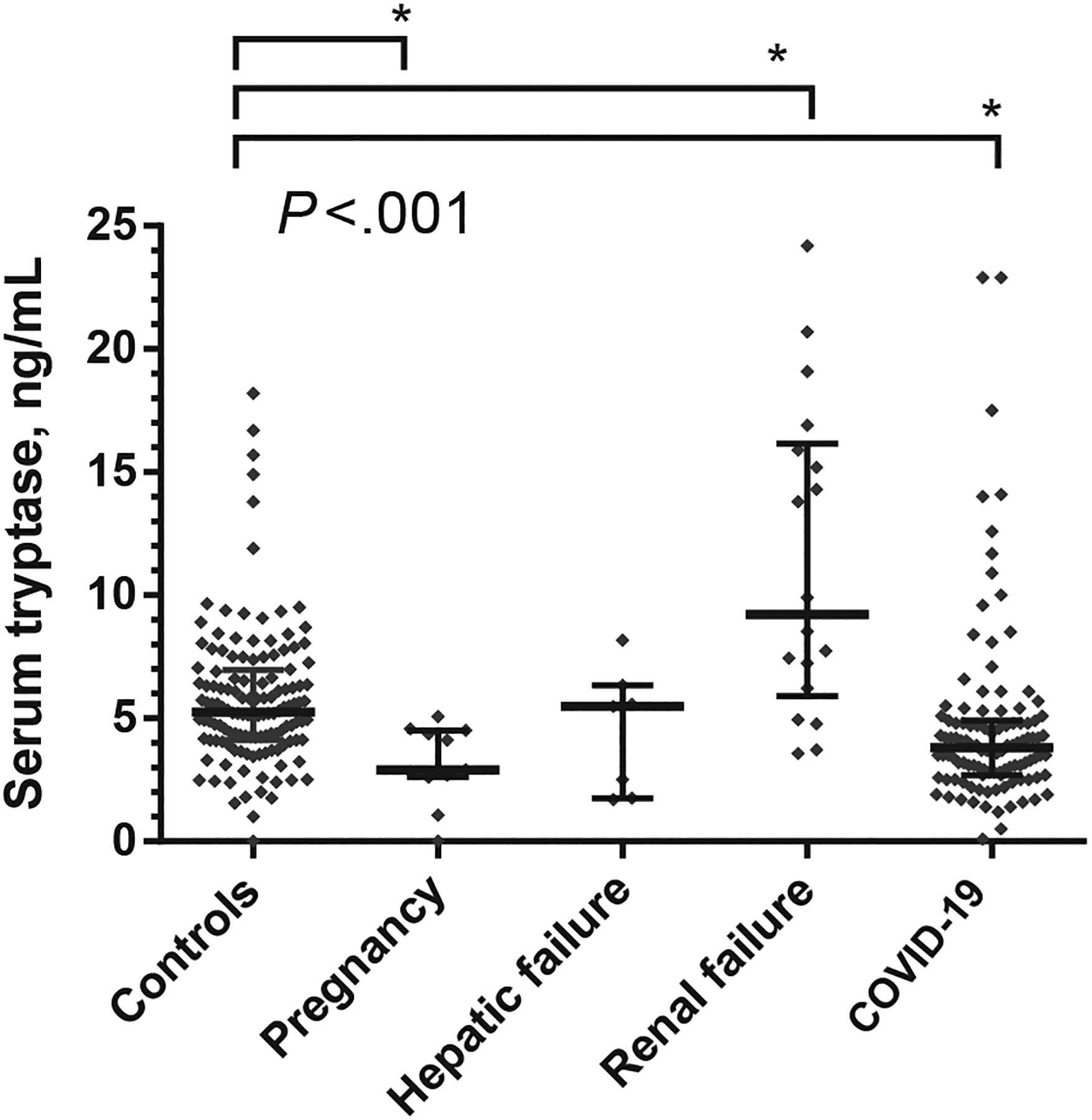
Serum tryptase levels in various groups of patients. Serum tryptase levels were measured in asymptomatic patients with hepatic failure (severe liver disease with clearly impaired liver function, n = 7), renal failure (n = 18), or coronavirus disease 2019 (COVID-19) infection (n = 110). In addition, serum tryptase levels were obtained in apparently healthy controls (n = 132) and pregnant women (n = 11). Tryptase genotyping was not performed in any of the patient or control groups. *P < .05 as assessed by analysis of variance.

A number of different conditions may be associated with a higher (or lower) BST level (Figure 2 and Table I).^30–36,42–49^ As mentioned, the most prevalent underlying etiology is HαT, an autosomal dominant genetic trait defined by an increased number of *TPSAB1* gene copies encoding alpha tryptase.^33,37,38^ Hereditary alpha tryptasemia is detected in about 4% to 7.5% of the population in the Western world.^47,48,50^ Most individuals with HαT present with a slightly to moderately elevated BST level (15–30 ng/mL).^37,38,47,48,50,51^ It has also been demonstrated that the serum tryptase levels correlate with the total copy numbers of the *TPSAB1* gene in these cases.^48,52^ However, HαT-positive persons may also present with a lower tryptase level (≤8 ng/mL^53^) or a rather high tryptase level (up to and rarely over 150 ng/mL). Those with a very high tryptase exhibit multiple *TPSAB1* replications.^48,51,52^

Clinically, most individuals with HαT do not have symptoms that had previously been associated with this trait.^48,50^ However, HαT has also been reported in patients who have experienced severe anaphylactic reactions, especially when a concomitant allergy and/or clonal MC disease are also present.^47,48,51^ Conversely, the occurrence of severe symptoms in individual cases can be predicted neither by the extra copy numbers of the *TPSAB1* gene nor by the basal tryptase level.

In patients with severely impaired kidney function, BST levels are often (slightly) elevated over the individualś baseline (Figure 2).^45,46,49^ In patients with chronic inflammatory diseases such as chronic infections, the basal tryptase level may increase, especially when chronic inflammation leads to MC activation and MC expansion in local organ sites. For example, slightly elevated basal tryptase levels may be found in a subset of patients with certain worm infections (eg, filariasis^49^).

The BST levels are also elevated in many patients with myeloid neoplasms, including a group of myelodysplastic syndromes (MDS), some with a myeloproliferative neoplasm (MPN), a subset of patients with chronic myeloid leukemia, about 30% to 40% of patients with acute myeloid leukemia, and some with MDS/MPN overlap disease.^42,44,49,54–56^ It is worth noting that, in most of these cases, no extra copy numbers of the *TPSAB1* gene are found and that, unlike in SM, the prevalence of HαT in these disorders is the same as in healthy controls.^48^ Patients with chronic eosinophilic leukemia and other eosinophil-rich neoplasms with a rearranged *PDGFR* fusion gene may also present with an elevated basal tryptase.^36,49,55,57^

**FIGURE 3.**
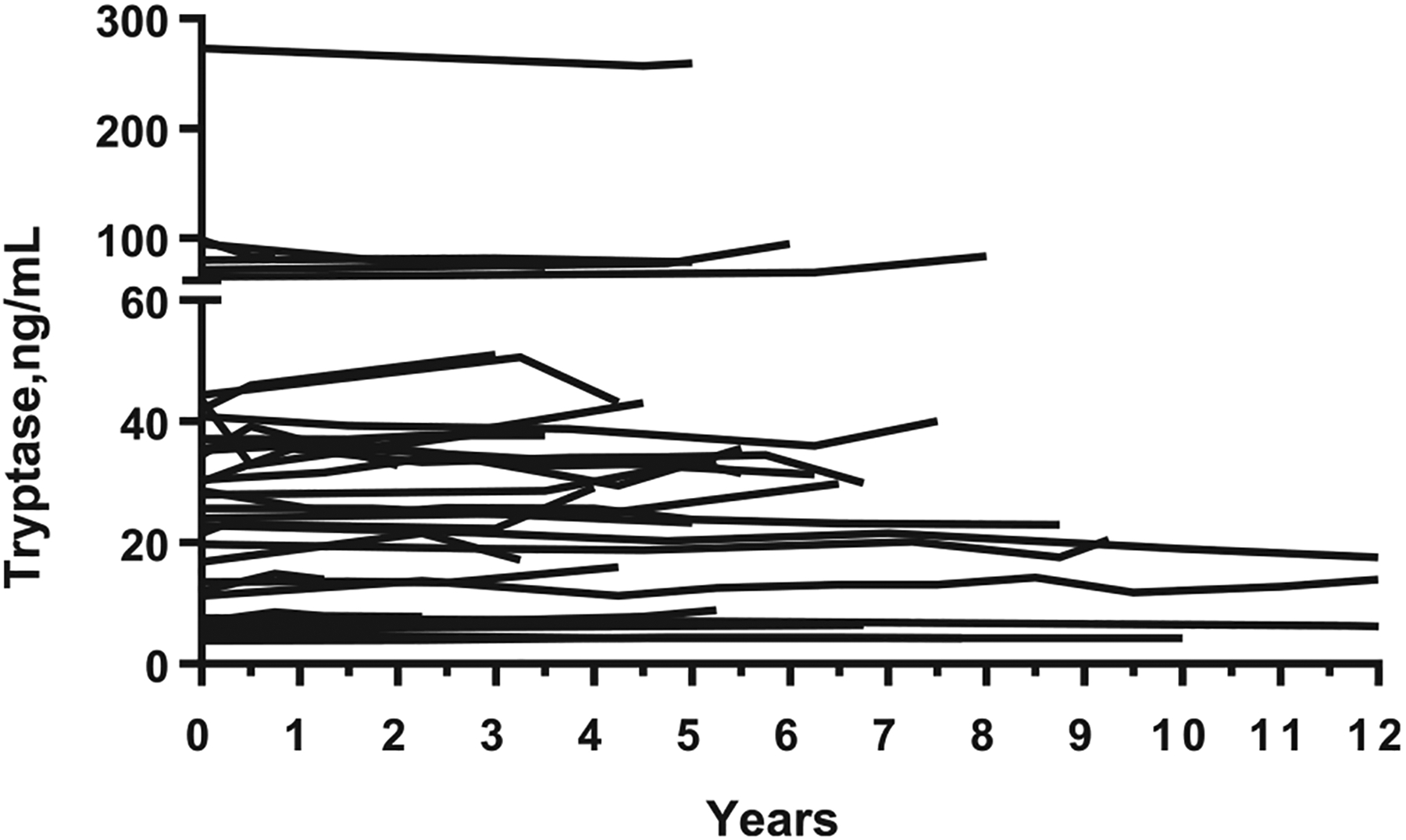
Longitudinal surveillance of BST concentrations in patients with mastocytosis. The BST concentrations were measured in 47 asymptomatic patients with BM mastocytosis, indolent SM, or smoldering SM at various time periods (median observation time 5 y). In all patients, the course of disease was stable and no severe mediator-related symptoms were recorded. Tryptase levels were measured by a commercial fluoroimmune enzyme assay as reported.^42–44,52^

Finally, elevated serum tryptase levels are found in the vast majority of patients with SM and correlate with the MC burden in these patients. Since 2001, a tryptase level of 20 ng/mL or higher counts as a minor diagnostic World Health Organization criterion of SM.^58–62^ The level of basal tryptase also correlates with the type and aggressiveness of SM.^43,59–61^ In particular, in most patients with smoldering SM or advanced SM (including aggressive SM and MC leukemia), markedly or even massively elevated BST levels are recorded.^43,58–62^ The highest levels are detectable in patients with MC leukemia. However, sometimes, very high serum tryptase levels are also found in indolent or smoldering systemic mastocytosis, especially when the MC burden is high and/or HαT is also present. Table I provides a summary of causes of an elevated basal tryptase level. An important aspect is that basal tryptase levels are usually stable and remain in the same range in most patients with nonadvanced SM over years (Figure 3).^43,61^ However, in some of these patients, tryptase levels increase or decrease, especially when the disease progresses or cytoreductive therapy is administered.^43,61,63^

**FIGURE 4.**
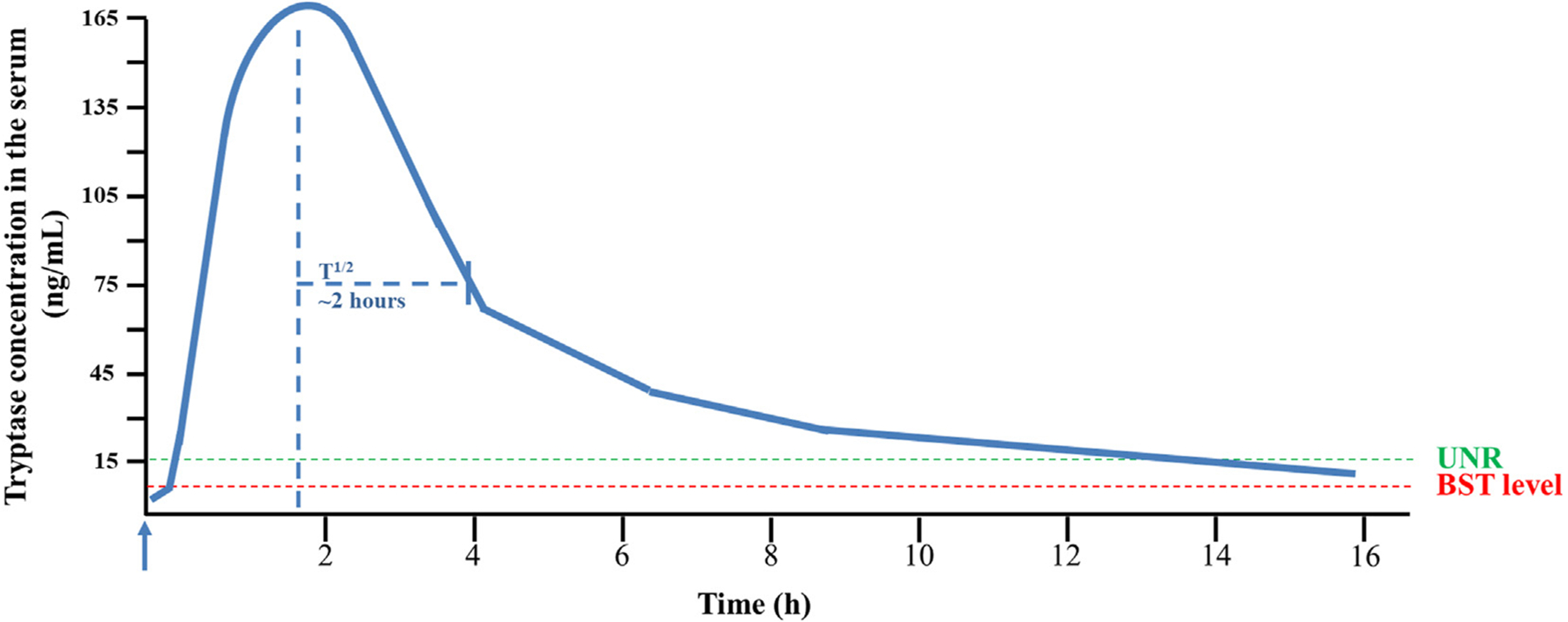
Kinetics of serum tryptase level during an anaphylactic reaction. A patient sensitized to Hymenoptera (wasp) venom was rechallenged by exposure to wasp venom (wasp sting). Just after reexposure to the allergen (blue arrow), tryptase is rapidly released from MCs, leading to a peak serum tryptase increase at about 1–2 h after the event. The half-life (T½) of tryptase in serum is approximately 2 h. Serum tryptase levels return to baseline within 24 h after resolution of all symptoms. The BSTwas measured before the anaphylactic event (red dashed line). *UNR*, proposed upper normal range threshold (15 ng/mL) (green dashed line).

During an anaphylactic reaction, MC activation and secretion of tryptase usually lead to a transient (often massive) increase in tryptase over the individualś baseline level (Figure 4). Therefore, it is important to measure the BST level in a symptom-free interval, which is 2 or more days after resolution of all symptoms of an anaphylactic reaction or other event related to systemic MC activation (Figure 4).

### Intra-individual variation of serum tryptase levels and event-related increase during a massive systemic anaphylactic reaction

As mentioned previously, the basal tryptase levels in individual donors (patient or healthy donor) are relatively consistent when tested repeatedly over time under stable and identical conditions (Figure 3).^31,32,39–43^ Even in patients with a very high basal tryptase level, the intra-individual range of basal tryptase often remains rather stable over time (Figure 3). However, as mentioned, there are a number of reasons for variations in basal tryptase levels, such as a decrease in kidney function, concomitant medications such as glucocorticoids, or a myeloid neoplasm.^31–37,42–48^ In such myeloid neoplasms (MDS, MPN, chronic myeloid leukemia), basal tryptase levels may be initially elevated and may rise further when the numbers of tryptasepositive, neoplastic cells (MCs, basophils, or lineage-committed blasts) increase due to progression.^42–44,49,54–56,63^ In patients receiving corticosteroids (systemic or topical), BST levels may decrease.^64–66^ For example, Schlag et al^66^ reported that, in patients with eosinophilic esophagitis, topical therapy with fluticasone (500 μg twice daily) resulted in a decrease of tryptase levels from 4.7 ng/mL to 3.8 ng/mL. In general, such drug effects may be an issue in patients with anaphylaxis in which event-related tryptase levels and post-event (basal) tryptase levels should be compared to define whether MCAS criteria are met. When the patient is still under corticosteroid therapy (but did not receive such drugs before and at the time of anaphylaxis), it may be reasonable to wait to retest for basal tryptase until corticosteroids are discontinued (Table II).

Over the past few years, there has been a debate about the stability and intradonor variability of BST levels.^67,68^ Indeed, in some of the studies, the intrapatient variability of tryptase was found to be substantial.^68^ In other studies, basal tryptase levels were found to be rather consistent over time in individual adults (Figure 3).^42,43,54^ The same has been described for children, although some variability in tryptase levels was found.^69^

Overall, the currently used serum tryptase test (fluoroimmune-enzyme assay) is robust, and the variation seen in the test results in individual donors (when tested over time) is usually not relevant clinically, provided that the conditions are stable and preanalytical issues are avoided. Table II provides recommendations for preanalytical measures and issues to address in the evaluation of the BST level in healthy donors and patients with MC neoplasms. When these preanalytical aspects and issues are taken into account, basal tryptase levels should be rather stable over time provided that the overall situation and conditions remain stable.

During an anaphylactic reaction, serum tryptase levels may increase over the individualś baseline level (Figure 4). The magnitude of the increase depends on several variables, including the severity of the reaction, time lapse between the blood draw and the reaction, type and number of organs involved, type of allergy, presence of comorbidities, and use and type of prophylactic therapy. When the reaction is massive and systemic (at least 2 organ systems involved), tryptase levels increase substantially (Figure 4). In these patients, MCAS is diagnosed when all MCAS criteria are met.^21–24^ These criteria include (1) typical clinical symptoms (usually in the form of anaphylaxis involving at least 2 organ systems), (2) an increase of the serum tryptase level to at least 120% + 2 ng/mL of the individualś baseline (or increase in other MC mediators), and (3) a response of the symptomatology to drugs targeting mediator effects (eg, receptors of these mediators, such as histamine receptors), mediator production, or MC activation (Table III).^21–24^ All 3 MCAS criteria must be fulfilled to diagnose MCAS in such a patient (Table III). Baseline tryptase levels should be measured at least 48 hours after (but not before) complete resolution of all clinical symptoms in these patients. An important aspect is that MCAS can be diagnosed in patients with clonal MC disorders including SM (primary MCAS), but also in patients with an IgE-dependent allergy (or other reactive disease) without evidence of a clonal MC disease (secondary MCAS).^21–24^

### The clinical impact of HαT: is HαT *per se* a silent genetic trait or indicative of a defined disease or pathology?

When defining a normal range for BST, a key question is whether HαT is a pathological condition (disease) or a genetic variant (trait) that may predispose to a certain symptomatology or acts as a modifier of other conditions. Based on previous and more recent data, HαT *per se* may not induce specific symptoms or a clinical syndrome.^50^ Moreover, no definitive correlation between HαT and a known specific reactive disease or allergy has been described to date. Conversely, the prevalence of HαT-positive individuals is higher in patients with SM (12.2%–20%) and those with idiopathic anaphylaxis (17%) compared with controls (4%–7.5%).^47,48,50,70^ The same holds true for patients with nonclonal (secondary) MCAS. In addition, HαT-positive patients suffering from SM and/or an IgE-dependent allergy have been described to have a higher likelihood to develop severe mediator-related symptoms during an allergic reaction than patients without HαT.^47,48^

From all these observations, the HαT trait may be regarded as a predisposition or modifier of certain clinical phenotypes. However, based upon current evidence, HαT *per se* should not be considered a disease-defining feature or pathology. Moreover, given that HαT may have been selected for in human evolution, one could speculate that this genetic trait may even impart some positive effects to our species.^71^ All in all, we believe that asymptomatic cases with HαT should be regarded as healthy individuals but not as patients. This is of major importance, because the range of normal serum tryptase should, therefore, also include some (4%–7.5%) asymptomatic individuals with HαT. In other words, the threshold to delineate normal from elevated basal tryptase levels in healthy subjects should be established independent of the HαT status.

### Previously proposed normal range of serum tryptase: overview and critical review

There is an ongoing debate about the normal range, especially the upper normal limit, of basal serum (total) tryptase measured in healthy individuals. In all recent studies, total tryptase values were measured by the same fluoroimmune-enzyme assay.^31,39^ It is generally accepted that the lower limit of detection in this assay is about 1 ng/mL (some individuals have even lower or undetectable tryptase levels that is not relevant clinically). In an initial unpublished study of the provider, the range of BST measured in 126 healthy individuals was 1.0 to 11.4 ng/mL (ImmunoCAP Tryptase Fluoroenzymeimmunoassay. Directions for Use 52–5679-US/03, October, 2019. Phadia AB). The upper limit of 11.4 ng/mL was determined by calculating the 95th percentile (95% confidence interval) of tryptase, indicating that only a few donors had tryptase levels beyond 12 ng/mL which is remarkable and may be explained by the fact that (1) only a few cases with HαT were included and/or (2) most donors with HαT (that were included) had a tryptase level below 11.4 ng/mL. In sub-sequent studies, the upper limit of normal tryptase was reported to amount to 8.23 ng/mL,^40^ 14 ng/mL,^41^ or 15 ng/mL.^42–44,54^ The different cut-off levels in these studies may be explained by a different statistical approach, by random variations in the fractions of HαT-positive individuals in these control cohorts, by age or ethnic factors, and/or by the fact that asymptomatic donors with a known allergy were either excluded or included in these analyses. Taken together, the physiological range of tryptase detected in all these studies, regardless of the genetic background or other features of the test population, ranged between 1 and 15 ng/mL.

An interesting aspect is that there are 2 distinct groups of individuals that present with lower or higher basal tryptase in these studies, indicating a bimodal distribution. In one group, tryptase levels clustered at around 4 to 11 ng/mL, and in a second, smaller group, tryptase levels were found to range between 12 and 20 ng/mL (Figure 2).^43,44,49,52^ Later, the second group was found to consist almost entirely of asymptomatic cases with HαT.^72^ It is important that this second group of healthy controls must not be neglected when determining a normal range of basal tryptase for daily practice. Rather, when establishing an internal reference range (healthy adults) in a routine laboratory, the control group should be checked for the following features: (1) normal kidney function, (2) no laboratory-based features or clinical signs or symptoms of an underlying hematological, MC-related, or allergic disorder, (3) no recent (past 6 h) intake of food or medications, and (4) presence of about 5% individuals with (suspected or known) HαT, as demonstrated by a tryptase level over 12 ng/mL (otherwise unexplained) and/or by a genetic test.

### Proposed lower limit of BST to exclude the presence of HαT-positive individuals in clinical practice

Because elevated serum tryptase levels are detected in most HαT-positive cases, the question is whether a lower threshold level can be recommended and used to exclude the presence of HαT with some certainty. When excluding most individuals with HαT, the normal tryptase range appears to be 1.0 to 11.4 ng/mL.^52,72^ However, some of the HαT-positive cases have low basal tryptase levels, ranging between 8 and 11.3 ng/mL, or even lower tryptase levels. Therefore, we recommend applying 8 ng/mL as a threshold to exclude a majority of healthy individuals carrying extra copy numbers of the *TPSAB1* gene (Table IV). This definition is important for clinical practice: in fact, testing for HαT should primarily be considered at a basal tryptase level of 8 ng/mL or greater, even if some cases with HαT may present with an even lower tryptase level (<8 ng/mL).^53,67^ Otherwise, this range is not relevant clinically, and it is important to understand that many healthy adults without HαT (and without any other etiology related to an increased tryptase) still present with a BST level of greater than 8 ng/mL or even over 11.4 ng/mL. In other words, a tryptase level of greater than 8 ng/mL (or even > 11.4 ng/mL) cannot be used as solid indicator (marker) of HαT in healthy or symptomatic individuals.

It is also worth noting that, in children and younger adults, serum tryptase levels are modestly lower than in older adults.^49,69,73,74^ In 1 study, serum tryptase levels in unselected healthy controls ranged between 0 and 15 ng/mL in younger individuals aged 10 to 30 years and between 4 and 16 ng/mL in adults older than 50 years.^49^ Tryptase levels also show a positive correlation with the body mass index and obesity,^75,76^ although this correlation was not identified in children.^77^

Finally, although serum tryptase levels appear to be rather stable in most patients with SM and also in most healthy controls under stable conditions, some studies have suggested that there is a certain intra-individual variation in the basal tryptase level when measuring repeatedly over time in healthy controls or patients.^42–44,50,68,73^ As mentioned previously, the intrapatient variability of serum tryptase can be minimized by taking pre-analytical caveats and issues into account.

### Proposed reference value of basal tryptase for daily clinical practice including (healthy) individuals with HαT

Based on the data reviewed previously and the discussion in our consensus group, we propose that the range for normal basal tryptase to be used in daily clinical practice (encompassing also asymptomatic cases with HαT) should be 1 to 15 ng/mL. This range covers all previously proposed ranges, all groups of healthy controls, including asymptomatic HαT-positive individuals, and also the 95% confidence interval calculated on the basis of 2 SDs in published cohorts.^31,39–44,49,54,55^ For daily practice, this normal range would help to avoid unnecessary investigations by caregivers and unnecessary anxiety in patients. Conversely, a normal tryptase level (<15 and even < 11.4 ng/mL) does not exclude SM, a myeloid neoplasm, or HαT with certainty. Therefore, a normal tryptase level cannot be applied as a major exclusion criterion for such conditions. Another important point is that the recommended threshold for delineating between normal and elevated is not identical to the threshold used to recommend an HαT test for genetic classification. Rather, our faculty recommends that tryptase genotyping should be considered when HαT is suspected (eg, patients with unexplained and/or severe anaphylaxis especially in patients with SM or a known allergy) and the basal tryptase level is at least 8 ng/mL. Sometimes, the test may even be recommended in a symptomatic patient with a tryptase level below 8 ng/mL.^53,67^

Finally, we propose that the BST levels should be classified as (1) normal lower range in which HαT is rather unlikely (1.0–8.0 ng/mL), (2) normal after excluding most (>90%) of the HαT-positive cases (1–11.4 ng/mL), (3) clinically normal, including asymptomatic controls with HαT (1–15 ng/mL), (4) hypertryptasemia (15.1–200 ng/mL), and (5) excessive hypertryptasemia (>200 ng/mL) (Table IV). It is important to state that hypertryptasemia and excessive hypertryptasemia are not a final diagnosis but have to be further examined regarding the underlying etiology. It is also important to note that most individuals with HαT have 10 to 30 ng/mL tryptase, but in a few families (with 10–15 extra *TPSAB1* copies), basal tryptase levels over 150 ng/mL may be found. Finally, we confirm that the threshold level of basal tryptase of 20 ng/mL, used as a minor criterion of SM, remains valid (Table IV).

### When should tryptase genotyping (HαT test) be recommended?

It is generally accepted that tryptase genotyping is an emerging genetic parameter that should be included as an integral component in the clinical evaluation of patients with suspected MC diseases (including SM and MC activation disorders) and related diagnostic algorithms.^24,62,67,73,75,78^ From a clinical point of view, knowledge about HαT is essential for many patients, both for estimating the risk of more severe anaphylaxis in certain populations and for estimating the probability that the patient is suffering from an underlying SM. Typical indications for a HαT test in clinical practice are summarized in Table V. The intention to determine the copy number status of the *TPSAB1* gene in individual cases depends on (1) the BST level, (2) clinical symptoms at presentation and in the case history (including family history), and (3) the age of the patient. Because extra copy numbers of *TPSAB1* are primarily detected in individuals with a serum tryptase level of at least 8 ng/mL, testing for HαT is usually recommended when the basal tryptase level is 8 ng/mL or greater.^37,38,67^ However, cases of HαT with a basal tryptase lower than 8 ng/mL (sometimes as low as 6.5 ng/mL or even lower) have been described.^53,67^ By contrast, in most patients with a tryptase level greater than 15 ng/mL, tryptase genotyping should be performed, unless the patient is asymptomatic and/or has a known underlying disease that explains the tryptase elevation (nephropathy, myeloid neoplasm, or MC neoplasm). For example, in an 80-year-old patient with an MDS who has no MC mediator-related symptoms and a stable basal tryptase level of 27 ng/mL, we would not recommend a genetic test. By contrast, in a 30-year-old patient with the same tryptase level who is suffering from recurrent severe mediator-induced symptoms, tryptase genotyping should be considered. If HαT testing is negative, the patientś peripheral blood leukocytes should then be tested for KIT D816V and the patient should be considered for a bone marrow (BM) biopsy to rule out SM and other BM neoplasms. If the HαT test is positive, no BM biopsy may be required, unless KIT D816V is also detected in blood leukocytes and/or other indicators of a coexisting SM or other BM neoplasm are present. It is important to know that the presence of HαT does not rule out SM. Rather, in symptomatic cases with SM and in SM cases in which tryptase levels are relatively high compared with the infiltration grade of the BM with neoplastic MC (eg, serum tryptase 120 ng/mL; BM infiltration grade 10%), tryptase genotyping should be considered because the likelihood of HαT is very high.^48^

A proposal to test for HαT may also arise from the family history, especially when several family members report severe systemic mediator symptoms and clinically relevant allergies. Another indication is the absence of evidence for an underlying disease responsible for the elevated tryptase (eg, nephropathy or a myeloid neoplasm) in clearly symptomatic patients suggesting MC activation. In asymptomatic donors and in most patients presenting with a number of other symptoms, including dysautonomia, certain connective tissue abnormalities, or irritable bowel syndrome, there is generally no indication to test basal tryptase levels or the tryptase gene status.

### Integrating tryptase and HαT in diagnostic algorithms

The BST level is a useful screening parameter in (1) patients with suspected SM, (2) patients with another suspected myeloid or eosinophil neoplasm, (3) patients with an MC activation-related disease (MCAS), and (4) patients with suspected or known HαT (eg, familial predisposition to severe anaphylaxis or MCAS).^30–32,39–44,47–56^ When the tryptase level is clearly elevated (>15 ng/mL) and no signs for a hematological neoplasm (including SM) are found in initial investigations, the patientś leukocytes are examined for the presence of a KIT D816V mutation and the presence of HαT.^24,62,71^ When KIT D816V is detected and/or HαT is not found, a BM investigation is usually recommended, and the final diagnosis may be indolent or advanced SM.^24,62,78–80^ In other patients with detectable KIT D816V, the diagnosis may be BM mastocytosis or, in case of skin lesions, cutaneous mastocytosis (CM). However, in those with BM mastocytosis and CM, tryptase levels are often below 20 ng/mL or even normal. When MCAS criteria and KIT D816V are detected, a monoclonal MCAS (MCAS with clonal MC) may be diagnosed even if the criteria for SM or CM are not met.

An important aspect is that in HαT-positive cases with suspected or known SM, BST levels should be corrected for HαT.^52,62,67^ Although the optimal way of correction is still under debate, one suggested approach is to divide the tryptase value by 1 + the extra copy numbers of the alpha tryptase gene.^62^ In some individuals, this may lead to a reclassification from SM to CM (because of lack of a third minor SM criterion) or from smoldering SM to indolent systemic mastocytosis (because a B-finding is no longer demonstrable).

In patients with suspected MCAS, it is important to confirm MCAS by applying MCAS criteria that (as mentioned earlier) include demonstration of a diagnostic increase (120% + 2 ng/mL) in tryptase over the individualś baseline during an anaphylactic event.^21–24,78^ When MCAS is confirmed, it is equally important to take the basal tryptase level into account: in those with a tryptase level lower than 8 ng/mL, genetic testing for HαT is not justified unless other family members are also known to suffer from symptoms of MC activation. In MCAS patients with a tryptase level of 8 ng/mL or greater, tryptase genotyping should be recommended.^67,78^ Finally, in symptomatic patients with a tryptase level greater than 15 ng/mL, the presence of HαT as well as the presence of the KIT mutation D816V should be tested for.^78–80^ This is important because patients who are suffering from SM, HαT, and an IgE-dependent allergy, have a high risk to develop MCAS. Although this association has been described repeatedly, larger prospective studies are needed to better characterize the potential impact of HαT in these patients. It is also worth noting that, in a small number of individuals with severe Hymenoptera venom allergy, a clonal MC disease may be detected even if the BST level is less than 8 ng/mL. Therefore, testing for HαT and for KIT D816V in clinical practice should always be based on the overall clinical situation in each case. It should be also stated that a negative test for HαT and for KIT D816V does not eliminate the diagnosis of SM.

### Concluding remarks and future perspectives

The BST is an extremely reliable and clinically important biomarker reflecting the total body burden of normal MC (non-neoplastic conditions) and of neoplastic cells producing tryptase (MC, immature basophils, and MC- or basophil-committed progenitors) in certain myeloid neoplasms. The assay exhibits excellent stability, precision, accuracy, and specificity. The intra-individual variability is usually low provided that the test is performed in an event-free interval and under stable and identical clinical conditions. The lower limit of basal tryptase below which only a few HαT-positive cases are detected, is 8 ng/mL although exceptional HαT cases with even lower tryptase levels have been identified. However, when including asymptomatic individuals with HαT, the normal range of basal tryptase is up to 15 ng/mL. This range should be applied in daily practice to avoid unnecessary referrals and investigations, and unnecessary fears in affected individuals.

## Figures and Tables

**TABLE I.** Etiologies associated with an elevated BST level

Etiologies	Mechanism	Expected range of BST*
HαT	Extra copy numbers of the *TPSAB1* (alpha tryptase) gene; autosomal dominant trait	8–50 ng/mL
Severe kidney damage (renal failure)	Not known—possible etiologies: reactive mast cell hyperplasia or altered enzyme metabolism	10–50 ng/mL
Obesity (increased body mass)	Not known	10–25 ng/mL
Chronic inflammatory disease processes and some chronic infections†	Expansion and/or persistent activation of tissue MCs	5–25 ng/mL
SM including MC leukemia	Increase and expansion of neoplastic MCs secreting tryptase, including alpha- and beta-tryptase	20–200 ng/mL
Myelomastocytic leukemia	Increase and expansion of immature neoplastic MCs secreting tryptase	15–50 ng/mL
Eosinophil neoplasms with *PDGFR, FGFR*, or other TK receptor rearrangements (MLN-TK)‡	Increase and expansion of clonal neoplastic MCs secreting tryptase	10–50 ng/mL
Chronic eosinophilic leukemia (CEL)	Increase and expansion of neoplastic MCs secreting tryptase	15–50 ng/mL
CML (chronic or accelerated phase)	Increase in immature leukemic basophil granulocytes or MC precursors secreting alpha-tryptase	15–50 ng/mL
Tryptase + AML	Increase in tryptase-producing AML blasts	20–100 ng/mL
Other myeloid neoplasms	Increase in tryptase-producing myeloid blast cells, progenitor cells, MCs, and/or basophils	10–50 ng/mL

*AML*, Acute myeloid leukemia; *CEL*, chronic eosinophilic leukemia; *CML*, chronic myeloid leukemia; *FGFR*, fibroblast growth factor receptor; *MLN*-*TK*, myeloid or lymphoid neoplasms with a TK gene fusion; *PDGFR*, platelet-derived growth factor receptor; *TK*, tyrosine kinase gene fusion.

* Expected tryptase range in which most affected individuals (patients) will be detected.

† Examples for chronic infections with elevated BST levels are certain helminth infections (filariasis).^49^

‡ In myeloid or lymphoid neoplasms with a TK gene fusion (MLN-TK), an increase in clonal MCs is often detected.

**TABLE II.** Pre-analytical measures and considerations to avoid potential issues in the evaluation of the BST level in daily practice

Fasting blood draw—especially in patients with a known allergy or an MCAS.
Blood should be drawn under stress-free conditions.
No severe mediator-related symptoms or signs of overt anaphylaxis during the past 2 d.
After collection of blood, serum should be obtained by centrifugation and used or stored within 24 h.
Serum can be stored at −20°C or at −80°C for several years. When prepared for testing, tryptase levels should be measured within 12 h after thawing.
Certain drugs, including mast cell stabilizers, KIT-targeting drugs, or corticosteroids may cause a decrease in serum tryptase levels. Such drug effects must be taken into account, for example, when tryptase levels have to be compared before and after an anaphylactic event to explore whether MCAS criteria are met.*

* Example: A patient has an elevated tryptase level during an anaphylactic event and received a corticosteroid after blood was drawn. If some days later (after the event), the patient is still being treated with corticosteroids, it may be reasonable to wait until the drug has been discontinued and only then to again draw blood to test for the basal tryptase level.

**TABLE III.** The diagnostic criteria of MCAS*

1.	Typical clinical signs of severe, recurrent (episodic) systemic MC activation involving at least 2 organ systems (skin, pulmonary, gastrointestinal, cardiovascular, naso-ocular) is present: typical manifestation = severe anaphylaxis.
2.	Involvement of MCs is documented by biochemical studies: preferred marker: increase in serum tryptase level from the individual’s baseline to 120% + 2 ng/mL†
3.	Response of symptoms to therapy with MC-stabilizing agents, drugs directed against MC mediator production or drugs blocking mediator release or the effects of MC-derived mediators.‡

*PGD*_*2*_, Prostaglandin D_2_.

* All 3 MCAS criteria (1 + 2 + 3) must be fulfilled to call a condition MCAS.

† Other MC-derived markers of MC activation that have been recommended: 24-h urinary histamine metabolites or PGD_2_ metabolites. These markers can also be employed but are less specific than the increase of tryptase levels. However, these alternative markers are recommended when the tryptase test is not available or is inconclusive.

‡ Example: histamine receptor blockers.

**TABLE IV.** Proposed classification of tryptase ranges for clinical practice and research

Proposed term of tryptase range	BST range
Normal range where HαT is rather unlikely*	1.0–8.0 ng/mL*
Normal range when excluding most individuals with HαT†	1.0–11.4 ng/mL†
Clinically meaningful normal range that includes (asymptomatic) individuals with HαT	1.0–15.0 ng/mL
Hypertryptasemia‡	15.1–200.0 ng/mL
Excessive hypertryptasemia‡	>200.0 ng/mL

* In this range, the likelihood to detect cases with HαT defined by *TPSAB1* extra copy numbers, is rather low. However, in a few patients with HαT, serum tryptase levels are below 8 ng/mL.

† Individuals (healthy or patients) with a normal copy number of the alpha tryptase (*TPSAB1)* gene. This range is also the normal range of BST proposed by the company providing the fluoroimmune enzyme assay.

‡ Hypertryptasemia and excessive hypertryptasemia are not a final diagnosis but have to be further examined regarding the underlying etiology. In this regard, it is also worth noting that most individuals with HαT have 10–30 ng/mL tryptase, but in a few families (>10 extra *TPSAB1* copies) basal tryptase levels may be > 150 ng/mL or even > 200 ng/mL. Levels > 200 ng/mL are also reached in some patients with advanced SM or acute myeloid leukemia. In addition a tryptase level > 200 ng/mL also defines the so-called B-finding in SM, a sign of massive expansion of the MC lineage in patients with SM.

**TABLE V.** Common indications for a HαT test in clinical practice*

Recurrent signs of MC activation of unknown etiology (eg, idiopathic anaphylaxis)
Familial clustering of signs of systemic MC activation (eg, anaphylaxis or MCAS in several family members)
Markedly elevated serum tryptase level (>15 ng/mL) without a known underlying myeloid neoplasm or mastocytosis
Suspected SM without KIT D816V but with a clearly elevated serum tryptase level
SM with relatively high basal tryptase compared with MC infiltration grade in the BM
SM with severe mediator-related symptoms (with or without a known allergy) or MCAS
Suspected or known idiopathic or secondary MCAS

* In all these indications, a HαT test is usually only justified when the BST level is at least 8 ng/mL.

## Abbreviations used

AIM: American Initiative in Mast Cell Diseases
BM: Bone marrow
BST: Basal serum tryptase
CM: Cutaneous mastocytosis
ECNM: European Competence Network on Mastocytosis
FcεRI: High, affinity receptor for the Fc region of immunoglobulin E
HαT: Hereditary alpha tryptasemia
IgE: Immunoglobulin E
ISM: Indolent systemic mastocytosis
MC(s): Mast cell(s)
MCAS: Mast cell activation syndrome
MDS: Myelodysplastic syndrome
MPN: Myeloproliferative neoplasm
SM: Systemic mastocytosis
SSM: Smoldering systemic mastocytosis
